# Nation Building and Social Signaling in Southern Ontario: A.D. 1350–1650

**DOI:** 10.1371/journal.pone.0156178

**Published:** 2016-05-25

**Authors:** John P. Hart, Termeh Shafie, Jennifer Birch, Susan Dermarkar, Ronald F. Williamson

**Affiliations:** 1 Research and Collections Division, New York State Museum, 3140 Cultural Education Center, Albany, NY, 12230, United States of America; 2 University of Konstanz, Department of Computer & Information Science, Universitätsstraße 10, 78464, Konstanz, Germany; 3 Department of Anthropology, 250A Baldwin Hall, Jackson Street, Athens, GA, 30602–1619, United States of America; 4 Department of Anthropology, University of Toronto at Mississauga, 3359 Mississauga Road, Mississauga, Ontario, L5L 1C6, Canada; 5 Archaeological Services Inc., 528 Bathurst Street, Toronto, Ontario, M5S 2P9, Canada; University of Toronto Mississauga, CANADA

## Abstract

Pottery is a mainstay of archaeological analysis worldwide. Often, high proportions of the pottery recovered from a given site are decorated in some manner. In northern Iroquoia, late pre-contact pottery and early contact decoration commonly occur on collars—thick bands of clay that encircle a pot and extend several centimeters down from the lip. These decorations constitute signals that conveyed information about a pot’s user(s). In southern Ontario the period A.D. 1350 to 1650 witnessed substantial changes in socio-political and settlement systems that included population movement, coalescence of formerly separate communities into large villages and towns, waxing and waning of regional strife, the formation of nations, and finally the development of three confederacies that each occupied distinct, constricted areas. Social network analysis demonstrates that signaling practices changed to reflect these regional patterns. Networks become more consolidated through time ultimately resulting in a “small world” network with small degrees of separation between sites reflecting the integration of communities within and between the three confederacies.

## Introduction

When present in the archaeological record, pottery is a mainstay of archaeological research worldwide. Pots and fragments thereof are subjected to a wide range of analyses to help build narratives of past human behaviors (e.g., [1,2]). Of particular note are the decorations that often occur on high proportions of pots at any given locale. These decorations, whether impressed into the clay itself through incisions, stamping or other means, or added to the surface through paint or other additive process, are frequently among the traits or the sole trait used to define archaeological taxa, which in turn are frequently associated with ethnicity. There have been few studies that seek to understand how pottery decoration changes in relation to trends in regional socio-political and settlement systems established through independent lines of evidence (e.g., [3]). In northern Iroquoia, comprising much of New York, southern Ontario and southern Quebec, changes in pottery during the last several centuries before European incursions are often associated with presumed trajectories of settlements leading to ethnic groups within territories recorded by early European chroniclers (e.g., [4–7]).

In his seminal publication MacNeish [4] described a large series of pottery types for northern Iroquoia that largely remain in use today. His type descriptions took into account historical ethnic territories, and he posited a dendritic model of ethnogenesis from what we now know is ca. 1000 years ago to historical ethnic groups. Using this framework many archaeologists working in northern Iroquoia have associated temporal and spatial variations in pottery with ethnicity. However, recent social network analyses (SNA) of pottery decoration in northern Iroquoia have challenged the assumption that pottery decoration reflects ethnic identity correlated with geographically-circumscribed territories [8,9]. In these analyses, collar decorative motifs are interpreted as active signals that convey information about pottery makers and users. The resulting networks reflect social signaling between village populations. Rather than distinct clusters within traditional ethnic territories, network ties between villages crosscut these territories, often at great distances. In fact, there is little correlation between physical distance and collar motif assemblage similarity [8]. It remains unclear, however, how pottery decoration and social signaling changed as the result of regional socio-political and settlement system trends.

Southern Ontario provides a unique opportunity to explore this topic. As a result of several decades of extensive archaeological investigations in compliance with cultural heritage laws and regulations in advance of major construction projects, combined with earlier and contemporaneous work done by various universities and governmental agencies, there is a robust archaeological record [10–13].

**Fig 1:**
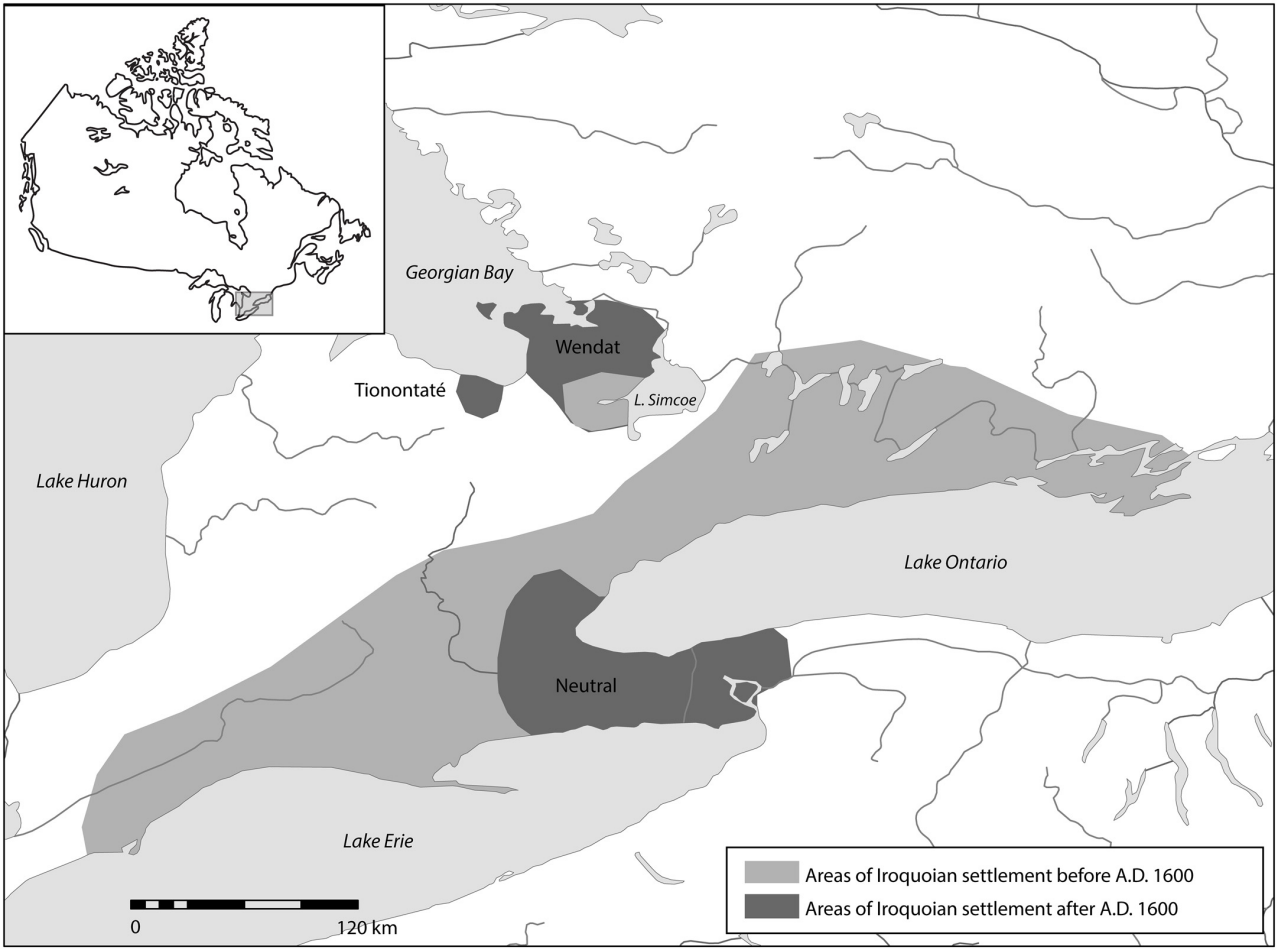
Location of precontact Iroquoian site distributions and historically-documented confederacies.

The fact that Iroquoian villages were occupied for an average of 10–40 years [10,11,14] and only rarely re-occupied means that each site represents a snapshot of the activities of a single generation. When villages were relocated, it was usually within a few kilometers and in the same drainage, although longer migrations also took place. The result is an archaeological record of site relocation sequences that represents multiple centuries of activity by contiguous community groups. This record includes a detailed three-century history of socio-political and settlement system change from ca. A.D. 1350 to 1650. During these centuries, populations in small dispersed villages along the north shores of Lake Ontario and Lake Erie coalesced into larger villages and towns in various river basins, formed politically-independent nations, and finally formed consolidated populations west of Lake Ontario and south of Georgian Bay. These areas were the home of three confederacies encountered by Europeans in the early seventeenth century and known historically as the Neutral, Petun (or Tionontaté) and Huron (or Wendat), respectively (Fig 1). The Wendat and Tionontaté were closely allied and shared many cultural characteristics, making their ancestral sites virtually indistinguishable prior to A.D. 1600.

In this article SNA of 125 archaeological sites using similarity values calculated from assemblage counts of decorative motif categories demonstrates changes in signaling networks that map onto the aforementioned regional socio-political and settlement system trends—aggregation into coalescent communities, the development of inter-community alliances and formalization of those alliances into nations, and finally the formation of political confederacies.

### Geopolitical change in south-central Ontario, ca. A.D. 1350–1650

Regional shifts in settlement systems and political alliances in south-central Ontario between A.D. 1350 and 1650, resulting in the historical Neutral, Tionontaté (Petun), and Wendat (Huron) Confederacies, are presented in Table 1 based on recent summaries by Birch [10] and Williamson [13]. At ca. A.D. 1350, Iroquoian communities in southern Ontario were living in sedentary villages, having fully committed to agricultural economies. Recent isotopic analyses by Pfeiffer et al. [15,16] indicate that by the fourteenth century the average contribution of maize to diets was some 50 percent. Between ca. A.D. 1300 and 1420, estimated ancestral Wendat-Tionontaté populations grew from ca. 10,000 to 24,000 [17] and it is likely that similar population growth was taking place in southwestern Ontario. This demographic growth resulted in an increase in the number of villages in south-central Ontario and the expansion of Iroquoian populations north into the Simcoe Uplands and eventually east into the Trent River Valley and even further to Prince Edward County. There is evidence for the development of practices and institutions that served to integrate a growing regional population [10,18,19]. By the early 1400s, ancestral Wendat, Tionontaté, and Neutral populations formed site clusters where multiple village communities shared drainage-based territories and, in some instances, contributed to combined ossuaries and associated mortuary ceremonialism [12,20].

**Table 1:** Socio-political and settlement system change in southern Ontario A.D. 1350–1650.

Period (A.D.)	Sociocultural characteristics
1600–1650	Consolidation of Neutral, Wendat, and Tionontaté confederacies in historic territories. Intensification of external conflict. Consolidated aggregate towns on frontiers, smaller village settlements in territory interiors.
1550–1600	Consolidation of nations. Consolidated aggregate towns (near Lakes Ontario and Erie), smaller village settlements (historic Wendake). Initiation of external conflict.
1500–1550	Initial nation formation. Consolidated aggregate towns. Palisaded, no evidence for expansions. Internal conflict in decline. Interregional interaction increases.
1450–1500	Coalescence. Formative aggregate towns, palisaded, with multiple palisade expansions. Some small villages remain. Internal conflict within the region.
1400–1450	Small villages clustered in major drainages.
1350–1400	Small to medium-sized, dispersed villages.

Beginning in the mid-1400s and continuing into the 1500s, many communities in south-central Ontario experienced seemingly simultaneous processes of endemic conflict and coalescence into large, palisaded towns [21]. It has been hypothesized that population growth, and possibly social circumscription and competition over hunting territories contributed in some way to an increase in violence and the formation of defensive communities [10,12,17,22]. These processes seem to have been more intense for populations located close to the north shore of Lake Ontario. It is unclear whether these same pressures were felt by populations in the Simcoe Uplands (historic Wendake).

**Fig 2:**
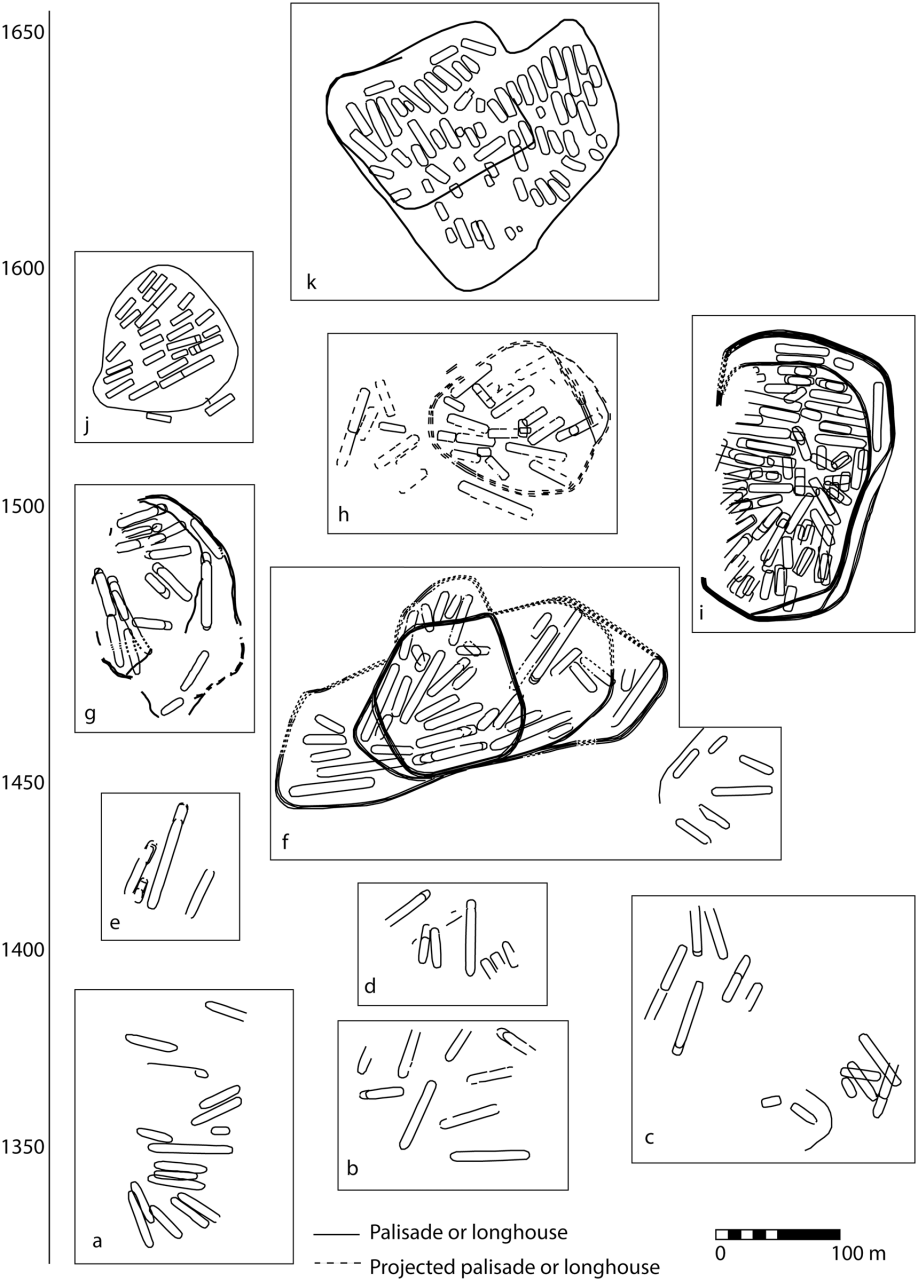
Selected Iroquoian site plans, ca. A.D. 1350–1650: a) Alexandra [25]; b) Robb [26]; c) Hope [27]; d) Over [28]; e) Baker [29]; f) Draper [30]; g) Keffer [31]; h) Kirche [32]; i) Mantle [33]; j) Benson [34]; k) Ball [35].

After ca. 1450, populations inhabiting drainage-based site clusters, together with populations originating further afield, aggregated into larger, coalescent village communities. Settlement locations favored defensible positions on elevated landforms away from riverine transportation corridors. Coalescent communities were surrounded by multi-row defensive palisades (Fig 2). The settlement plans of formative coalescent communities exhibit evidence of longhouse and palisade expansion and the addition of new clusters of longhouses, suggesting that coalescence occurred rapidly during the estimated 10–40 year average lifespan of villages. Butchered, burnt, and modified human bone occurs in significant quantities in village midden deposits after 1450 [23]. Post ca. 1500, the quantity of human remains in middens declines, although villages remain palisaded [10]. Archaeological and ethnohistoric evidence suggest that coalescence influenced the formation of politically and territorially coherent nations during the sixteenth century [10,13,24]. There is evidence that these political groupings were composed of multi-ethnic social units [8,9].

Gradual population movements to the north and east continued throughout the fifteenth and sixteenth centuries and seem to have involved sub-community groups (lineages and clans) as well as the relocation of entire communities, although the precise mechanisms underlying processes of polito- and ethnogenesis are only beginning to be understood. By the early seventeenth century, alliance-building and population movement resulted in the formation of the historically documented Neutral, Tionontaté, and Wendat confederacies [10,13,24,36] (Fig 1). For the Wendat and presumably Tionontaté, the process of confederation had its genesis in the gradual movement away from the north shore of Lake Ontario northward and consolidation in the Simcoe Uplands, south of Georgian Bay beginning in the late thirteenth century A.D. For the Neutral, this involved a contraction of their traditional territory in southwestern Ontario and relocation to their historical location at the western end of Lake Ontario. In the 1630s, smallpox and other epidemics took drastic tolls on these populations, with mortality rates reaching some 60 percent [37,38]. By 1650, Iroquois raids from the south aimed at replenishing their own depleted populations and control of lucrative European trade networks resulted in the incorporation or dispersal of all three Ontario Iroquoian confederacies.

### Ethnicity and signaling

Recent archaeological treatments of ethnogenesis have tended to focus on colonial contexts (e.g., [39–41]); the concept perhaps has less often been applied to internal processes of socio-political change. Traditional perspectives on ethnogenesis view it as a process of boundary making defined through inter- and intra-group negotiation [40,42]. However, ethnogenesis can also be a process of boundary removal, whereby previously distinct groups negotiate and adopt shared symbols and practices that extend ethnic boundaries. Weber [43] argued that ethnic identity is differentiated from kinship and culture by its role in forming a political community. In this way, social actors may manipulate markers of ethnic identity as they negotiate changing geopolitical landscapes. This is particularly important in current efforts to understand processes of coalescence and socio-political system changes among northern Iroquoian societies.

Blanton [44] has recently coupled Barth’s notions of ethnic boundaries to collective action theory (also see [45,46]). Blanton notes that a group’s system of visual signaling can provide archaeologists with information about the manners in which trust was generated among members of a collectively organized group. Members of such a group may have been socially distant from one another, but their new shared affiliation was expressed through “stylistic messages” (see [47]). As communities migrate into new areas, it is likely that signaling networks change, perhaps as the need to maintain old networks diminishes [48]. Of particular note is the concept of meta-identities [49]. In the coalescence of diverse populations new group identities develop as a result of increased interactions and the need to break down or merge previous social identities. Such meta-identities arise in coalescent communities through active invention or spontaneously through the repeated interaction of individuals from previously separate communities now living in closer proximity to one another [49]. Such meta-identities require the development of new signals or the reconfiguration of existing signals to reflect the mutual interests of new social entities.

Here we investigate the production of social relationships and the forms of signaling used to express those relationships [50]. While these relationships correspond in many ways to concepts of ethnicity and identity, they can be difficult to infer in archaeological contexts [51], [52]. These issues can be overcome by reframing ethnicity in terms of signaling networks [9] as they relate to processes of politogenesis—the formation of complex political organizations or systems.

### Signaling and northern Iroquoian pottery

**Fig 3:**
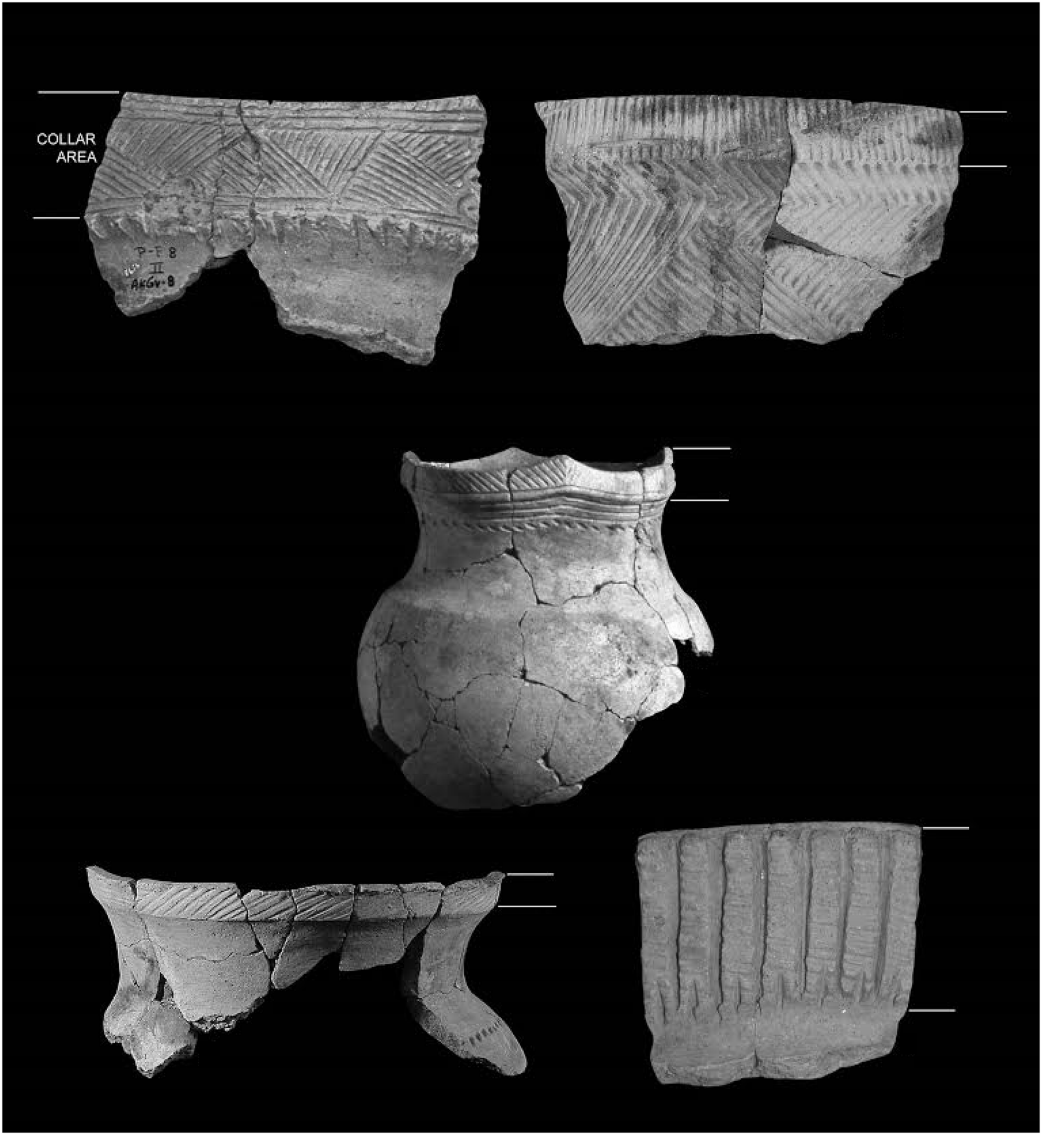
Examples of collar decoration on Ontario Iroquoian pottery.

Iroquoian pottery is characterized by collars—thickened bands of clay that extend around the pot and several centimeters down from the lip (Fig 3). These platforms are generally decorated with incised or stamped straight lines forming often complex geometric patterns in horizontal bands around the pot that are sometimes enhanced with punctations. Based on the more than 3000-year record of pottery production and use in northern Iroquoia [53], we know that neither collars nor decorations were needed for pots to function well as cooking vessels [8], [54]. The creation of the collar as a decorative platform and application of the decoration added unnecessary time and material costs to pottery production [8]. Added costs included extra clay and temper, the time needed for careful application/construction of heavy collars to thin-walled, unfired vessels, the time needed for planning and implementing decorations on the collar, and increased drying time and care needed in firing [9].

Iroquoian women were the primary producers of ceramic vessels [55]. These women were active participants in political affairs—they held councils, arranged marriages, elected and deposed leaders, and maintained the domestic economy [56–58]. As negotiators, consensus builders, and transmitters of skills, women’s power was exerted in domains not always considered to be explicitly political; domestic spaces were among those places where women exercised their political power [59]. Given that household interiors served as the locations of female domestic and political activities, collar decorations provided signals of network membership to those involved in such activities within the primary context of pottery use [59,60]. While other portions of vessels, such as the lip and neck were often decorated, unlike collars they were not always decorated throughout the period of time and space in question here. Additionally, collars were the most visible portion of a pot in both domestic and public contexts [61]. Decorative motifs on collars were readily decoded and comprehended by the viewer. As a result, collar motifs had high absolute and contextual visibility [62] and were, therefore, active signals [9]. These motifs mirrored or complemented signals visible in wider ranges of contexts such as tattoos, pipes, dress, hair styles and other forms of ornamentation [9,63]. These motifs, then, provide a means of exploring signaling networks and their changes through time and across critical hinge-points in the historical development of northern Iroquoian societies.

## Materials and Methods

Iroquoian potters combined a limited set of motif elements in dynamic ways to form more complex motifs and motif combinations which can be recorded in a variety of ways. Several collar-decoration coding schemes have been used by archaeologists working in northern Iroquoia (e.g., [6,7,64–66]). Given the geometrical nature of collar decorations, most of these schemes are interchangeable. We used Engelbrecht’s scheme [64,65], which employs combinations of attributes to form 28 decorative analytical motif categories. As in other schemes, each of Engelbrecht’s categories comprises a range of visually related possible combinations of decorative elements. The use of other schemes may result in at least somewhat different networks. However, the 28 categories capture more variation in motifs than do other schemes in use that rely on sometimes substantially fewer categories.

Undecorated collars, which comprise a 29th analytical category in Engelbrecht’s scheme, were not included in calculations described below. Perhaps such pots were used when no signaling was necessary or desired. We acknowledge that other portions of vessels, including lips, necks, collars, and bodies also included decorative attributes. Future analyses which incorporate additional attributes will undoubtedly augment the results of the present study. However, given the high visibility of collar decoration we believe it was a particularly salient medium for signaling practices and serves as a robust data set for social network analysis. Furthermore, we acknowledge that intra-site signaling practices may have varied within communities and among their constituent parts. However, we restrict our analyses to the inter-site signaling, treating individual settlements as nodes within regional networks (see e.g., [3]).

Included in our analysis are 125 sites, each with a minimum of 25 rims, resulting in the coding of 46,246 collar decorations. Collar decoration data from site reports, theses, and journal articles, and original coding forms in archives and made available by regional archaeologists, were re-coded using Engelbrecht’s scheme. Other assemblages were coded directly from collections (S1 Table). The resulting data were added to an existing database of codes [9] comprising site assemblage counts of each of the 28 decorative motif categories. This effort expanded the number of southern Ontario sites in the database from 33 to 125 (S1 Appendix).

Counts of decorative motif categories by site were used to calculate a Brainerd-Robinson (BR) similarity coefficient matrix. This is a form of city-block coefficient that is widely used in North American and in northern Iroquoian archaeology, specifically, to determine similarities between artifact assemblages (e.g., [6,67]).We used the Similarity and Distance Measures module of TFQA 5.0 [68] to generate the BR similarity matrix adjusting for sample size variation with the module’s Monte Carlo pairwise routine specifying 1000 trials and random seed generation from the clock. Motif assemblage evenness was calculated as Simpson’s *E*_*1/D*_ [69].

**Fig 4:**
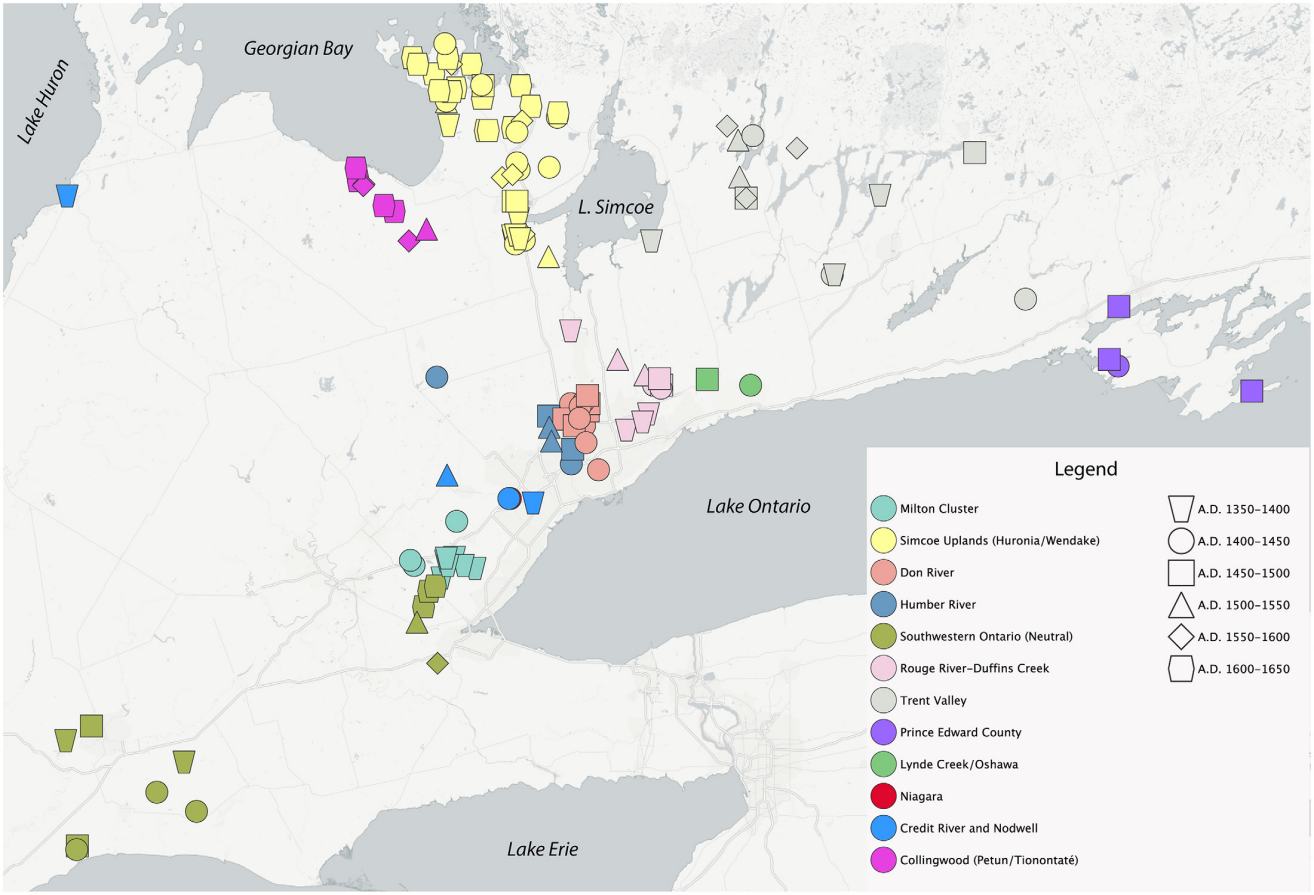
Distribution of sites used in the SNA.

Each site was assigned to one of six 50-year periods: (1) A.D. 1350–1400, (2) 1400–1450, (3) 1450–1500, (4) 1500–1550, (5) 1550–1600, and (6) 1600–1650. Temporal assignments were based on ceramic seriation, radiocarbon dates, and settlement patterns. Sites were also assigned to one of 12 geographical areas based on Lake Ontario watershed drainage, site cluster, or other physiographic features such as the Simcoe Uplands (Fig 4). Previous archaeological research along with ethnohistorical records of the early contact period suggests geographically based village clustering and sequences [10,11,13]. Groups represent site relocation sequences associated with one or more village-communities.

A GIS was developed to generate a straight-line distance matrix between all sites using ArcINFO v. 10.2. Site locations were established in the GIS using the Universal Transverse Mercator [UTM] coordinate system. Distances between sites were determined using the ArcINFO Point Distance Tool.

Networks were constructed for each two sequential time period sequence using the BR coefficients. These constructions take into account the potential effects of lagged responses of networks as communities moved to new locations and as different communities or members of separate communities coalesced into single villages. Pairs of nodes, or sites, were connected at a BR similarity threshold of 125, the minimum value needed to keep all nodes in the network connected with the exception of one extreme outlier. The networks were created in Visone [70]. A Backbone layout algorithm was used to untangle and identify the ties with the strongest influence on the network structure, thus also detecting natural grouping of the nodes [71]. Higher Backbone strength is shown with darker ties. The single- and sequential-period networks were analyzed using several descriptive network statistics. These included network density (Δ), defined as the ratio between the number of apparent ties to the number of possible ties; clustering coefficient (*CC*), defined as the tendency for nodes in the networks to cluster together; and average path length (*APL*), defined as the average number of steps from each node in a network to all others. *CC* values were normalized to account for different network sizes by calculating the mean of the local clustering coefficients by dividing the sum of each node’s coefficients by the total number of nodes in the network.

The E-I index [72,73] was used to determine how the centrality of nodes vary according to regional group for single- and sequential-period networks. This index decomposes common centrality indices with respect to a selected node attribute so that relative contributions to the centrality of a node given this attribute can be measured. In our analysis, this attribute was a node’s geographical group allowing each site’s propensity for within- and between-group connections to be determined, referred to as homophily and heterophily, respectively. This index is applied in an archaeological context to degree centrality [74] and is defined as the number of ties that a node has to other nodes, within-group (internal, homophily) and between-group (external, heterophily) in the network. We also calculated the index with closeness centrality, which is the extent to which a given node has short paths to all other nodes in the network. The most central node given these two measures, then, is the node with the most number of ties, and the node with the shortest geodesic distance to all other nodes. The E-I index for degree centrality formally is given as

$$\text{E-I index}=\frac{E-I}{E+I}$$

where *E* is the number of external edges between groups, and *I* is the number internal edges within groups for any given site. The measure varies between +1 and −1, where perfect heterophily is +1 and perfect homophily is −1. Although the formula above also applies for closeness centrality, its interpretation is reversed in that a positive E-I means homophily and negative E-I means heterophily.

The E-I index is not dependent on the network density; however, it is sensitive to varying group size and thus requires a normalization. Degree E-I was normalized as described in Everett and Borgatti [72] where the observed external and internal edges are divided by their respective maximum. Closeness E-I was normalized in a similar manner as regular closeness centrality. Given a connected network, closeness centrality is divided by a normalizing constant given as the maximum geodesic distance between two nodes in the network, that is *(n−1)*, where *n* is number of nodes in the network. Closeness E-I was normalized by dividing *E* and *I* with maximum distance between and within groups, respectively. This normalization of the E-I index is formally given as

$$\frac{\;\left(\frac{E}{n_{E}\left(n-1\right)}\right)-\left(\frac{I}{n_{E}\left(n-1\right)}\right)}{\;\left(\frac{E}{n_{E}\left(n-1\right)}\right)+\left(\frac{I}{n_{E}\left(n-1\right)}\right)}$$

where *n*_*E*_ is the number of nodes outside of the focal group, and *n*_*I*_ is the number of nodes within the focal group. An E-I value for each network as a whole was calculated as the sum of all site E-I values divided by the number of sites [74].

Analysis of Variance (ANOVA) was used to test differences and similarities between the measured E-I scores among the regional groups and other data for single- and sequential-period networks. The procedure compares the variance between and within group means to determine whether the groups are from the same or different populations.

Coefficient of variation (*cv*), which is calculated as the standard deviation of a sample divided by its mean, was used to compare variation in some motif percentages. The following equation was used to correct for sample size bias [75]:

$$cv^{*}\;=\;\left(1+\frac{1}{4n}\right)cv$$

where *n* is sample size.

## Results

### Visualizing networks

**Fig 5:**
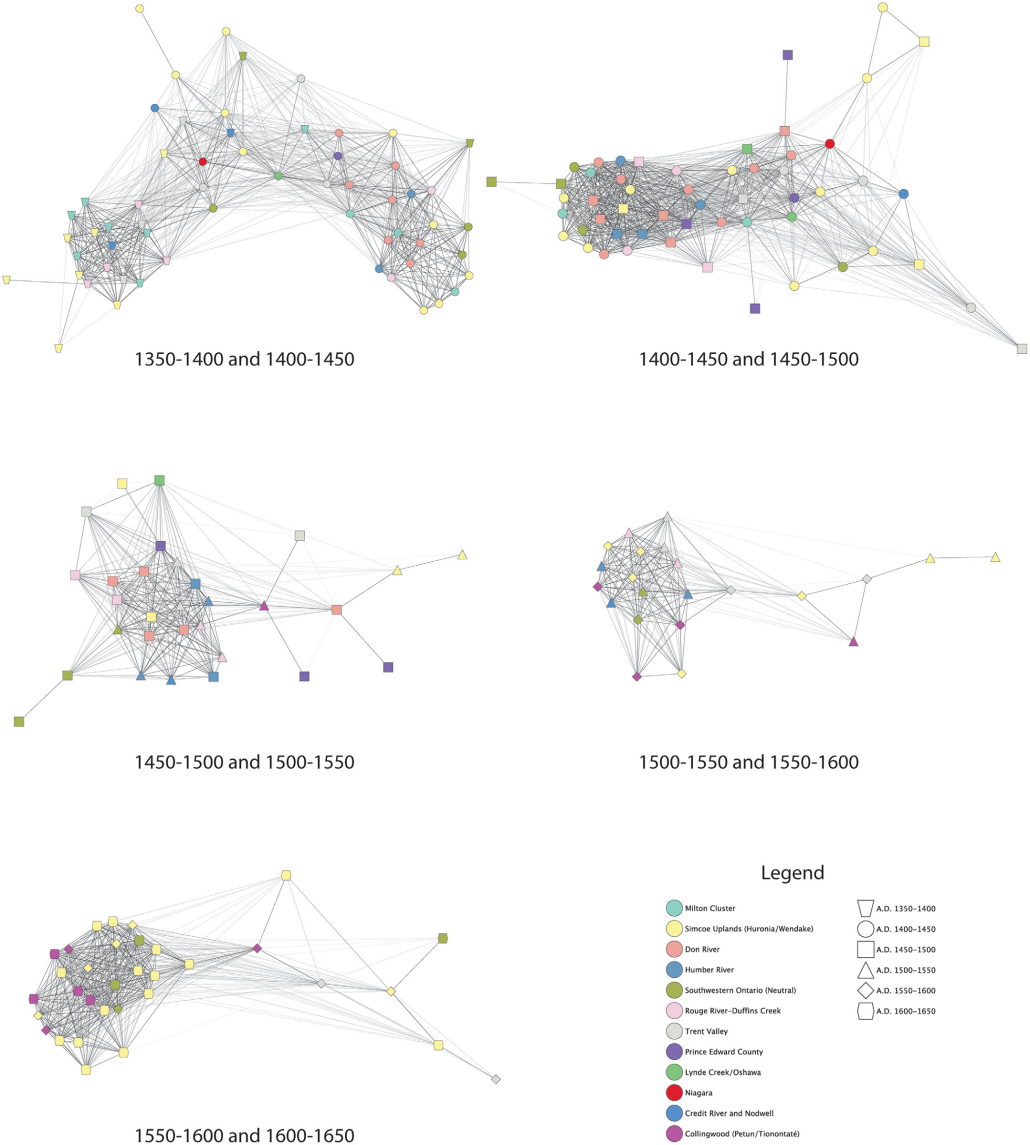
Networks of sequential periods.

**Fig 6:**
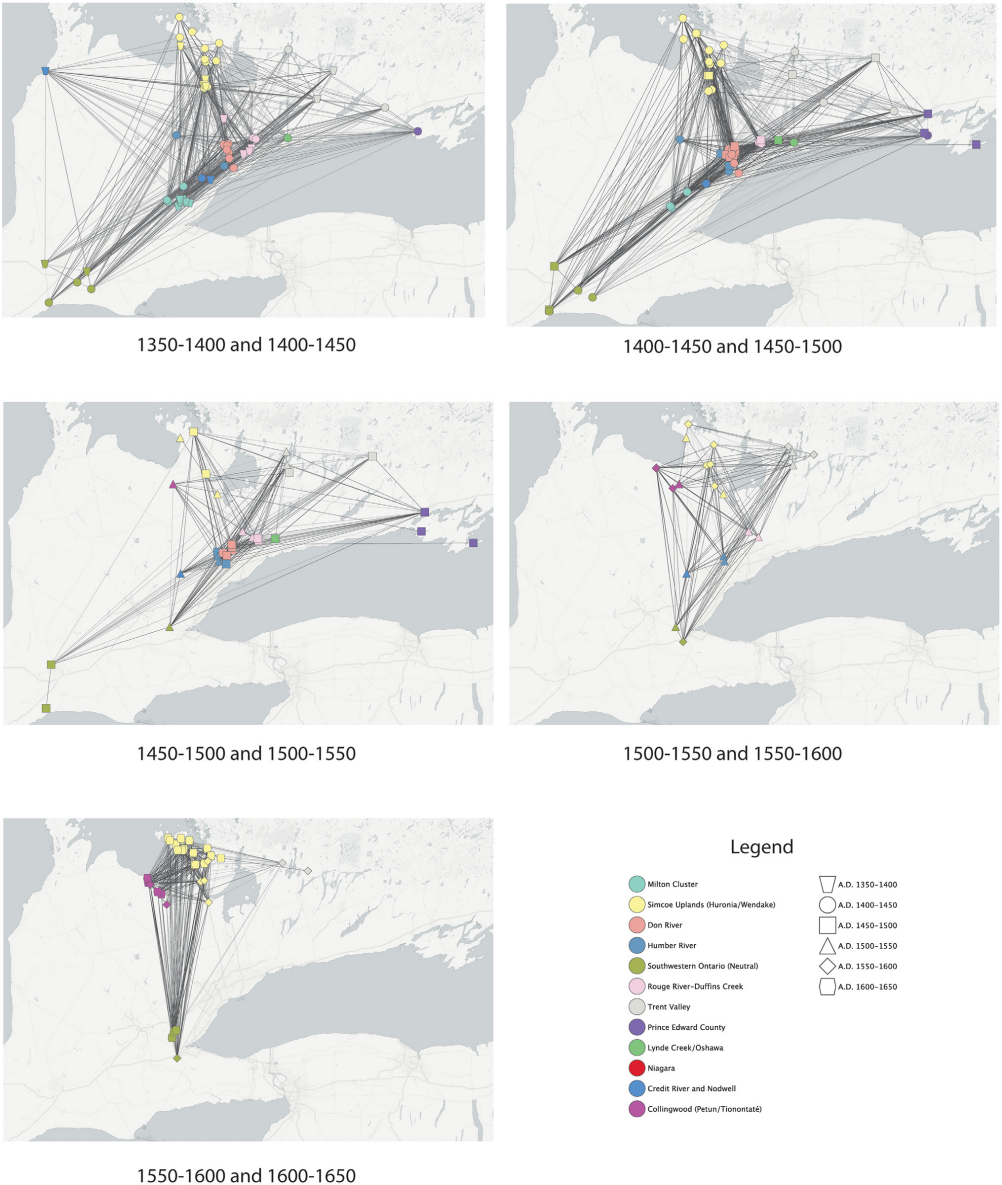
Networks of sequential periods with sites in geographical position.

Networks by two sequential time periods are presented in Fig 5, and networks with sites located in their geographical positions are presented in Fig 6. Apparent in each of these figures is that signaling networks transcend geographic subregions. While there are often strong ties among sites in any given area, there are also strong ties between sites separated by relatively great distances. In Fig 5, the two main clusters are defined by time period as opposed to distance. Both clusters consist of sites from different geographical regions. Frequently there are stronger ties between sites in different areas than between sites assigned to the same geographical group. Fig 6 illustrates strong ties between sites in the Simcoe Uplands and sites on the north shore of Lake Ontario in the 1350–1400 and 1400–1450 periods. These in turn have strong ties to sites north of Lake Erie.

These patterns repeat in the 1400–1450 and 1450–1500 network. Fig 5 shows that with a few exceptions, there is a lack of clear clustering of sites within geographical groups, with strong ties occurring between sites in different groups. Pairs of sites from the same locations within the main cluster do have strong ties, but each site is also strongly tied to sites from other areas. Sites in the Trent Valley are only found on the right side of the plot, along with more than half of the Simcoe Upland sites, suggesting that eastern populations, and some groups in the Simcoe Uplands, were not as strongly integrated into the main network as groups to the south and west. The geographical based plot in Fig 6 shows strong ties between some Simcoe Uplands and sites in the drainages north of Lake Ontario, while these in turn have strong ties to sites in southwestern Ontario. Sites in Prince Edward County have no or weak ties among themselves, but each has strong ties with sites to the west.

The 1450–1500 and 1500–1550 and A.D. 1500–1550 and 1550–1600 (Figs 5 and 6) networks correspond to the period of coalescence and conflict described above. Sites in the drainages north of Lake Ontario cluster together and are now more strongly connected to sites in the Trent Valley and the Collingwood/Tionontaté area. There remain strong ties between sites assigned to different geographical groups. In the 1450–1500 and 1500–1550 network, sites in southwestern Ontario are now more weakly tied to the main network. There are fewer direct ties between some sites in the Simcoe Uplands, sites in southwestern Ontario, and sites in Prince Edward County. In fact there are no strong ties between the two 1500–1550 Simcoe Upland sites and other sites from that period of time. In the 1500–1550 and 1550–1600 network, sites in southwestern Ontario are once again strongly tied into the main network, which is based on strong ties between sites near Lake Ontario, in the Trent Valley, and in the Collingwood/ Tionontaté area. The two 1450–1500 Simcoe County sites remain as outliers, with a single strong tie to a 1550–1600 Trent Valley site.

The A.D. 1550–1600 and 1600–1650 networks (Figs 5 and 6) show the consolidation of sites in the Simcoe Uplands (Wendat), the Collingwood area (Tionontaté), and west of Lake Ontario (Neutral). While these three groups are geographically and politically separate there are strong ties between sites assigned to different groups. As shown in Fig 5, some Neutral and Tionontaté sites cluster strongly with the majority of Wendat sites, while other Wendat and Neutral sites are outliers, along with one site in the Trent Valley. There also appear to be distinct subclusters of Wendat and Tionontaté sites. There are outlying sites to the right of the main cluster.

### Descriptive network statistics

Network density increases through time in both the single- and two-period networks (Table 2). For the two-period networks there is a monotonic increase in the clustering coefficient. For the single-period networks, there is no overall trend, but the highest value occurs in the 1600–1650 period. For the two-period networks there is a monotonic decrease in *APL*. For the single-period networks there is no overall trend, but the lowest value occurs in the 1600–1650 period. *APL* and *CC* follow the same trend given the density values. This results from the network density increasing, indicating that sites are more clustered and more easily reached from each other in the last time period. This is especially apparent in the two-period networks.

**Table 2:** Network statistics by time period(s).

Period	nodes(*n*)	ties(*m*)	Δ	*CC*^a^	*APL*	corr(BR, Dist)	corr(closeness E-I, degree E-I)	closenessE-I	degree E-I
Single Period Networks									
1350–1400	25	183	0.61	0.83 (0.82)	1.45	-0.21	-0.72	.087	-.087
1400–1450	34	333	0.59	0.77 (0.78)	1.47	-0.14	-0.68	.046	-.087
1450–1500	19	94	0.61	0.77 (0.78)	1.56	-0.52	-0.85	.132	-.035
1500–1550	11	35	0.64	0.89 (0.75)	1.51	-0.27	-0.94	.231	-.351
1550–1600	11	41	0.75	0.86 (0.88)	1.29	-0.30	-0.96	.134	-.200
1600–1650	22	191	0.83	0.93 (0.94)	1.17	-0.27	-0.81	.034	-.086
Sequential Period Networks									
1350–1450	59	712	0.42	0.70 (0.72)	1.75	-0.16	-0.71	.059	-.092
1400–1500	54	812	0.57	0.80 (0.81)	1.51	-0.26	-0.63	.080	-.124
1450–1550	30	256	0.59	0.88 (0.84)	1.49	-0.46	-0.69	.076	-.007
1500–1600	22	160	0.69	0.91 (0.83)	1.39	-0.28	-0.55	.019	-.071
1550–1650	33	421	0.89	0.91 (0.92)	1.21	-0.30	-0.93	.059	-.091

^a^Values in parentheses are not normalized.

### Geographical distances and BR coefficient

Central tendency values and ranges for geographical distance and BR similarity values are presented in Table 3. Following moderate increases in average and median distance between 1350–1400 and 1450–1500, there is a substantial drop in average distance from 1450–1500 to 1600–1650. BR similarity values are greatest and have the narrowest range in 1600–1650. The second highest BR values occur in 1500–1550. There is a high negative correlation between median (-0.881) and average (-0.728) BR values and geographic distance indicating that as distances decrease BR values increase. ANOVA indicates significant differences in geographical distance and BR for all periods (Table 4). ANOVA of sequential periods indicates significant differences for BR between 1350–1400 and 1400–1450, 1450–1500 and 1500–1550, and 1550–1600 and 1600–1650. For each of these pairs the second period has higher average values. For geographical distances there are significant differences between 1400–1450 and 1450–1500, 1450–1500 and 1500–1550 and 1550–1600 and 1600–1650. The value for 1450–1500 is higher than that for 1400–1450, while the later period in the other two pairs is lower. The correlations of BR similarity and geographical distance for all networks except 1450–1500 and 1450–1550 are negligible (Table 2). The 1450–1500 period has a moderately negative correlation and for 1450–1550 there is a low negative correlation.

**Table 3:** Geographic distance and BR similarity coefficient central tendency values and ranges by period.

	Geographic Distance (km)						BR similarity					
**Period**	1350–1400	1400–1450	1450–1500	1500–1550	1550–1600	1600–1650	1350–1400	1400–1450	1450–1500	1500–1550	1550–1600	1600–1650
**Mean**	88.7	95.2	110.9	71.0	70.6	52.6	122	128	126	135	125	151
**Median**	91.7	84.8	97.8	67.7	62.4	34.2	129	130	124	147	132	154
**Maximum**	277.1	326.8	358.7	162.7	176.9	154.0	180	190	181	185	184	191
**Minimum**	0.71	0.47	1.1	3.9	0.53	1.0	29	56	52	60	36	104

**Table 4:** ANOVA results for BR Values and Geographic Distance between Time Periods.

	BR		Distance	
Periods	*F*	*p-value*	*F*	*p-value*
All	33.08	.000	24.29	.000
1350–1400 and 1400–1450	4.00	.046	2.37	0.124
1400–1450 and 1450–1500	1.35	.245	7.48	0.006
1450–1500 and 1500–1550	4.03	.046	12.53	.000
1500–1550 and 1550–1600	2.35	.128	0.003	0.958
1550–1600 and 1600–1650	54.23	.000	6.906	0.009

These results indicate that there are significant differences in the geographic distances between sites for three of the five sequential pairings of single-period networks. These differences reflect changes in the regional distributions of sites. BR values track the changes in geographical distances in two instances, with higher BR values associated with decreased geographical distances between the 1450–1500 and 1500–1550 and the 1550–1600 and 1600–1650 periods. Central tendency values for BR and geographic distance by period have high correlations. However, within most single- and two-period networks BR values are not correlated with geographical distances suggesting little if any effect within specific networks. The only exception is 1450–1500 when there is a substantial increase in the central tendency values for geographic distances compared to 1400–1450 while there is little change BR central tendency values. On the whole, then, with decreasing distances between sites there is a trend for greater motif assemblage similarities. But, distances between sites are not correlated with assemblage similarities for most networks.

### Assemblage evenness and number of motifs

There is a trend for decreased motif assemblage evenness through time indicating that fewer motifs account for greater proportions of the assemblages (Table 5). There is also a trend for fewer average motifs per site through time, reaching a nadir in 1600–1650. ANOVA indicates that the differences between all time periods are significant for each of these variables (Table 6). By 1600–1650 the corpus of signals represented by collar motifs is smaller—on average communities relied on fewer motifs to signal their membership(s) in network(s). ANOVA of values for sequential time periods indicate differences for evenness between 1350–1400 and 1400–1450 and between 1550–1600 and 1600–1650. For number of motifs there are significant differences between 1350–1400 and 1400–1450 and between 1400–1450 and 1450–1500.

**Table 5:** Mean number of motifs and evenness by period with one standard deviation.

Period	No. motifs	Evenness
1350–1400	10.03±3.19	0.14±0.03
1400–1450	8.27±3.07	0.10±0.04
1400–1450	11.15±4.00	0.10±0.05
1450–1500	11.82±6.08	0.08±0.03
1550–1600	9.75±5.80	0.09±0.07
1600–1650	7.09±3.20	0.06±0.02

**Table 6:** ANOVA results for number of motifs and evenness.

Periods	No. Motifs		Evenness	
	*F*	*p-value*	*F*	*p-value*
All	3.90	.003	8.14	.0000
1350–1400 and 1400–1450	4.75	.033	18.04	.0001
1400–1450 and 1450–1500	8.89	.004	.34	.565
1450–1500 and 1500–1550	.15	.714	3.26	.081
1500–1550 and 1550–1600	.70	.413	.71	.410
1550–1600 and 1600–1650	3.01	.095	4.74	.037

The predominant motif category in all periods is 3 (Table 7), left or right oblique or vertical lines, what is typically referred to in the Ontario literature as “simples” [6]. As shown in Table 7 the percentage of this motif category increases substantially from 1350–1400 to 1400–1450 and then reaches its largest percentage in 1600–1650. The cv* is highest in 1350–1400, and lowest in 1600–1650. The increase of cv* in 1550–1600 is explained by two Tionontaté sites that have low percentages of this motif category. Removing these two sites from the calculations results in a mean approaching and a cv* that is lower than that of 1600–1650. In 1600–1650, the Tionontaté sites have values in the range of Wendat and Neutral sites.

**Table 7:** Mean, standard deviation (*σ*) and coefficient of variation (cv*) for Motif 3 by period.

Period	Mean	*σ*	cv*
1350–1400	30.8	13.6	44.1
1400–1450	62.0	18.2	29.6
1450–1500	57.3	15.8	27.9
1500–1550	65.5	18.7	29.3
1550–1600	66.0	23.4	36.2
1550–1600 adjusted	74.2	14.1	19.4
1600–1650	78.1	13.0	16.8

### Degree and closeness E-I

Pearson correlations between degree and closeness E-I are negative and become stronger through time (Table 2). This means that there are consistent results of homophily and heterophily in the two E-I indices. E-I values for the individual networks are presented in Table 2. The highest closeness E-I values occur in 1500–1550 and the lowest in 1600–1650. An ANOVA indicates significant differences for E-I closeness when all periods are considered (*F* = 4.52, *p* = .0009). ANOVA also indicates significant differences between 1550–1600 and 1600–1650 (*F* = 13.17, p = .001) when there is a decrease in the coefficient. ANOVA results indicate no significant differences between periods for degree E-I whether analyzed as a whole or in sequence.

**Fig 7:**
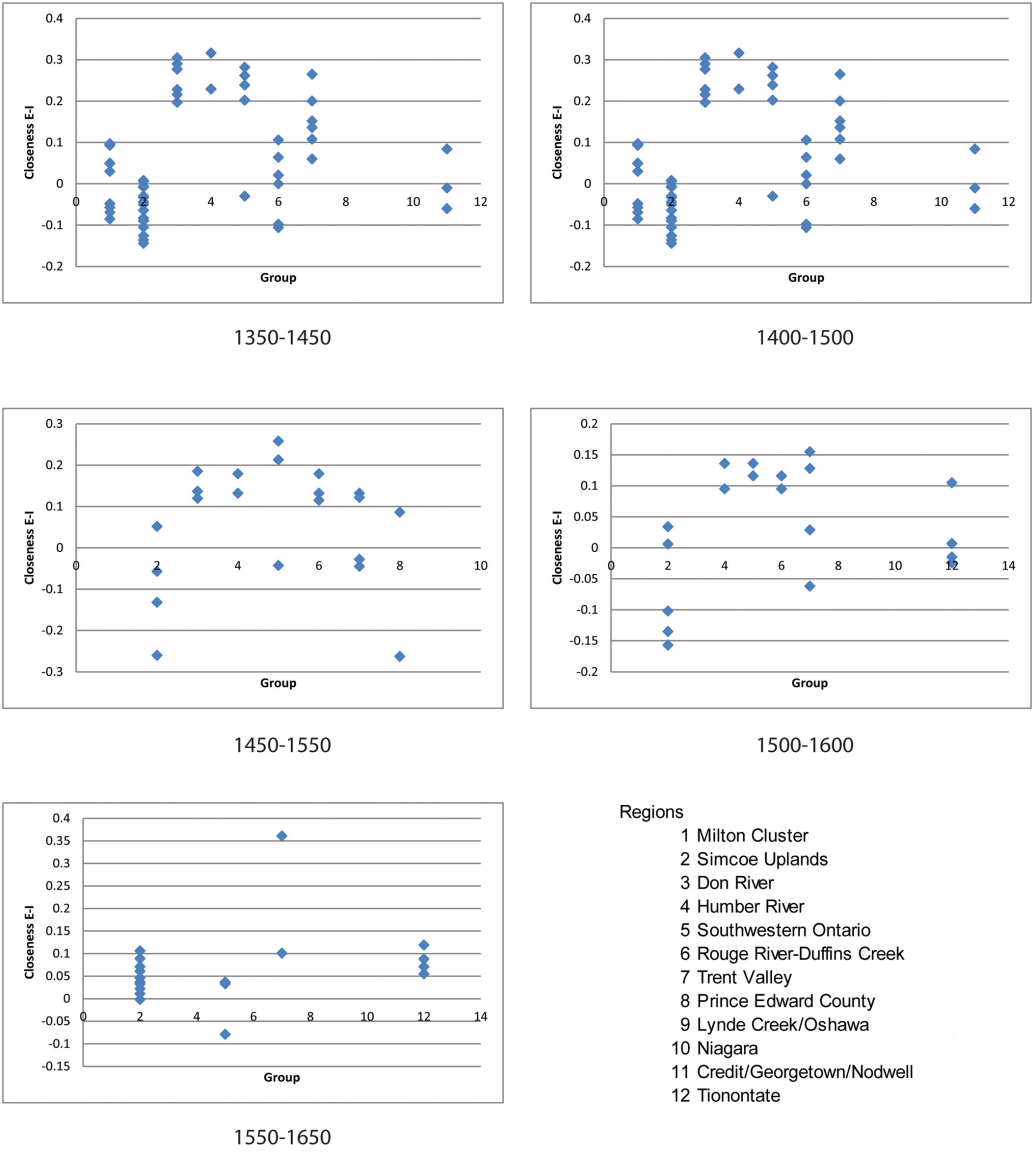
Sequential period closeness E-I values by group.

The two-period network values indicate that most of the E-I closeness values for sites in the Simcoe Uplands in networks 1350–1450 through 1500–1600 are negative or low positive, while those for other groups north of Lake Ontario are mostly positive (Fig 7). The Simcoe Upland sites in the 1550–1650 network have low-positive closeness E-I values. These results suggest that through most of the 300-year span of time the majority of the communities in the Simcoe Uplands had a greater emphasis on external signaling than did most of the communities in other geographically identified groups. An examination of individual time period networks indicates that the greatest emphasis on external signaling for the Simcoe Uplands communities was in 1400–1450, a time of continued migration into the region by communities and smaller social groups. In 1550–1650 there is a shift that suggests increased importance of internal signaling for the Simcoe Upland sites, coincident with the consolidation of Wendat populations in this area and confederacy formation.

## Discussion

While not all statistical trends are monotonic, each reflects the final consolidation of Wendat and Tionontaté communities south of Georgian Bay and Neutral communities on the west shore of Lake Ontario. Of particular note is the fact that despite negative correlations between the central tendency values for BR and geographic distance through time, there are negligible correlations between geographical distance and motif assemblage similarity for most periods. Also of note is the difference between early sites in the Simcoe Uplands and those of other geographical groups north of Lake Ontario, with the Simcoe Uplands sites having predominantly external signaling concerns while the others have predominantly internal signaling concerns.

During 1350–1400 the archaeological record consists of small, discrete village sites distributed widely across the landscape. This is also just after the initial expansion of Iroquoian populations into the Simcoe Uplands, which became the homeland of the historical Wendat confederacy. Strong ties between sites in the Simcoe Uplands and those in other areas may represent continued interaction with sending populations or the diffusion of signaling practices. In 1400–1450 villages remain small and shift to a settlement pattern clustered in drainage basins. While there are distinct geographical clusters of sites in the network plots during this time, distance was not a factor in determining signaling networks. Average distance between sites increases slightly between these two periods as does the average BR values, the latter of which is statistically significant. The 1350–1400 period is also when the average evenness value is highest, and evenness decreases significantly to the 1400–1450 period. These changes indicate that as settlement systems changed from dispersed villages to geographically distinct clusters of villages, signaling networks relied on fewer collar motifs. However, the networks were not focused on the village clusters but, rather, were widely dispersed, as reflected in the larger distances between villages, the slight decreases in network density and clustering coefficient and increase in *APL*, and E-I closeness and degree values near zero. The network dynamics of this period provide historical context for coalescence and conflict in the next period.

The 1450–1500 period was a time of coalescence characterized by the formation of larger, aggregated, palisaded towns with multiple house and palisade expansions. This was also a time of conflict within the region and beyond, with evidence for conflict extending through ca. 1550. Within this dataset, patterns of coalescence and conflict are clear on the north shore of Lake Ontario and in southwestern Ontario, although populations in the Simcoe Uplands may not have experienced the same pressures, perhaps reflected by their outlier positions in the 1500–1550 network (Fig 5). This period of regional strife is reflected in signaling networks by a significant increase in distances between sites, a moderately negative correlation between geographical distance and BR values, non-significant decrease in BR and closeness E-I values, and an increase in *APL*. The mean number of motifs per site increases significantly, while assemblage evenness remains constant, interpreted as a continued focus on a small number of motifs by resident potters, and an increase in very small proportions (>1%) of motifs potentially representing non-local residents or visitors [76]. These results suggest that as communities coalesced during a time of regional strife; there was a shift in signaling networks reflecting a greater emphasis on signaling within geographic clusters. This shift in signaling patterns may reflect a social strategy which included alliance-building between near neighbors, possibly related to communal defensive strategies.

An examination of the E-I indices by group indicates that in the first three time periods, sites in the Simcoe Uplands (historic Wendake) account for most of the heterophily values (Fig 7). For 1350–1400 these sites account for 3 of the 4 negative closeness E-I values, for 1400–1450, 7 of 11, and for 1450–1500, 1 of 2. These values indicate an external focus of signaling during the first three periods for the Simcoe Uplands site relative to other geographical groups. The only other geographical group with a dominant focus on external signaling is in historical Neutral territory during the 1400–1450 period when each of the three sites represented have negative closeness E-I values. Otherwise, the sites in our sample for the various geographical groups have positive values suggesting foci on internal signaling. These data indicate that there may have been multiple communities contributing to the colonization of the Simcoe-Uplands Iroquoian frontier. Externally-oriented closeness E-I values indicate that groups in this area retained connections to groups to the south as depicted in the network plots. This may be explained in part by the continued movement of population to the Simcoe Uplands throughout the temporal sequence, with population expansion occurring first to take advantage of new environmental niches and continuing later as the result of population pressure, conflict, and social circumscription as coalescent communities were galvanized into formative nations [11–13,18].

The 1500–1550 period is a time of consolidated, aggregate palisaded towns with less evidence for village expansions. Evidence for regional conflict declines, and there is a significant decrease in the distances between sites and a significant increase in BR values. There are increases in clustering coefficient and network density and decreases in *APL*. Degree and closeness E-I homophily peak during this 50-year period. This results largely by the positive values for the Simcoe Uplands sites. There is no change in the number of motifs per site and a non-significant decrease in assemblage evenness. All of this evidence suggests that there were consolidations in signaling networks continuing the trend initiated in the previous 50-year period. Networks were more internally focused, while maintaining ties between sites in different groups. These patterns may suggest that nation formation began as early as ca. 1500. By 1550–1600 the north shore of Lake Ontario was settled only by consolidated aggregate towns whereas both small and large village settlements are found in historic Wendake. This is also the period of initiation of external conflict. Network density increases from the previous period and *APL* decreases. The E-I values decrease, but continue to indicate homophily. There are no significant changes in either distances between sites or in BR values.

The 1600–1650 period is a time when archaeologists and early European chroniclers have inferred and observed, respectively, the existence of the Neutral, Wendat, and Tionontaté confederacies, the latter two of these having a close relationship. Network density and clustering coefficient reach their highest values, and *APL* its lowest value. There is a significant decrease in the average distance between sites and a significant increase in BR values. E-I values approach 0 indicating concerns with both internal and external signaling. The average number of motifs drops. Motif category 3 reaches its highest percentage during this period. These results indicate a consolidation of signaling networks with the formation of confederacies, but also demonstrate that the three confederacies did not have completely distinct signaling networks. This is best illustrated in Fig 5 in which sites from all three confederacies cluster together, with some outliers and in Fig 6, which clearly illustrates the strong ties that occur between sites belonging to the three confederacies. While the existence of alliances and neutral military positions between these three political groups has been previously recognized, the identification of strong network ties and signaling behavior between these politically and geographically distinct populations is a major contribution of this study. The structure of these interconnections is suggestive of a “small-world network” that increases information flow between nodes through both structured and random processes, resulting in a dense network structure that reduces the “degrees of separation” between nodes [77,78].

## Conclusions

Pottery design sequences typically have been used in northern Iroquoia to identify ethnic affiliations of pre-contact sites and to hypothesize population movements through time. Recent SNA of collar decorations across northern Iroquoia has questioned these traditional interpretations and their underlying principles. A theoretical context was developed that identified collar decorations as signals [9]. SNA of these signals suggest that rather than ethnic markers, the decorations signal inclusion in network memberships that crosscut traditional ethnic and political boundaries. With a very robust archaeological record that has produced evidence for major shifts in settlement patterns, regional coalescences of village populations, changes in regional strife, and ultimately the development of confederacies, southern Ontario is an excellent area to investigate how signaling networks adapt as a result of socio-political and settlement system changes.

Political apparatus and the political community may be distinct [79] and a coherent political body need not be ethnically homogeneous. As such, processes of affiliation and associated signaling patterns may link people with diverse origins and affiliations. Our results demonstrate that the communities living in southern Ontario from A.D. 1350 to 1650 were members of signaling networks that integrated the region—networks extended across the region throughout the 300 year time span. However, the networks also tracked major changes in socio-political and settlement systems. By the end of the sequence the networks are highly consolidated and integrated. This conforms to the movement of communities into geographically constrained areas and the formation of political confederacies.

As suggested by Clark [49], the ending network included a signal of meta-identity, in the form of simple collar decorations consisting of vertical or left or right oblique lines. These motifs served as regional identifying signals throughout the sequence, but especially after 1400. By 1550–1600 this motif became a meta-identifying signal and by 1600–1650 it was adopted by most communities in the three confederacies. Other motifs remained in use but only represent small percentages of assemblages. The adoption of uniform signaling practices can be seen as a social strategy [80] that helped to integrate Ontario Iroquoian communities and confederacies during the early contact era, a period marked by external conflict, depopulation, and rapid and profound social changes stemming from direct European contact.

Our results demonstrate that pottery decoration can be used to help elucidate changes in regional socio-political systems. Even in a highly integrated region like southern Ontario from A.D. 1350 to 1650, changes in networks based on collar decoration are evident. Archaeological applications of SNA have the potential to add new layers of understanding to regional systems through time.

## Supporting Information

S1 Appendix Data used in analyses. (XLSX)

S1 Table Sources of data used in motif category coding for SNA. (PDF)

## References

[1] Skibo JA. Understanding Pottery Function. New York: Springer-Verlag; 2013

[2] Rice PM. *Pottery Analysis*: *A Source Book*, Second Edition Chicago: The University of Chicago Press; 2015.

[3] Mills BJ, Clark JJ, Peeples MA, Haas WR, Roberts JM Jr, Hill JB, et al Transformation of social networks in the late pre-Hispanic US Southwest. *Proc Nat Acad Sci USA* 2013; 110: 5785–5790. doi: 10.1073/pnas.1219966110 23530201 10.1073/pnas.1219966110 PMC3625298

[4] MacNeish, RS. Iroquois pottery types: a technique for the study of Iroquois prehistory. National Museum of Canada Bulletin No. 124, Anthropological Series No. 31. Ottawa:National Museum of Canada; 1952

[5] Niemczychi MAP. The origin and development of the Seneca and Cayuga tribes of New York State Research Records No. 17. Rochester, New York: Rochester Museum and Science Center; 1984.

[6] Ramsden PG. A Refinement of Some Aspects of Huron Ceramic Analysis. Ottawa: National Museum of Canada;1977.

[7] Tuck JA. Onondaga Iroquois prehistory: a study in settlement archaeology. Syracuse: Syracuse University Press;1971.

[8] Hart JP. The effects of geographical distances on pottery assemblage similarities: a case study from northern Iroquoia. J Archaeol Sci. 2012; 39:128–134.

[9] Hart JP, Engelbrecht W. Northern Iroquoian ethnic evolution: a social network analysis. J Archaeol Meth Theor. 2012; 19:322–349.

[10] Birch J. Current research on the historical development of northern Iroquoian societies. J Archaeol Res. 2015; 23:263–323.

[11] Birch J, Williamson RF. The Mantle site: an archaeological history of an ancestral Wendat community. Lanham, Maryland: AltaMira Press; 2012

[12] Birch J, Williamson RF. Navigating ancestral landscapes in the northern Iroquoian world. J Anthrop Archaeol. 2015; 39:139–150.

[13] Williamson RF. The archaeological history of the Wendat to A.D. 1651: an overview. Ontario Archeol. 2014; 94:3–64.

[14] Warrick GA. Estimating Ontario Iroquoian village duration. *Man in the Northeast* 1988; 36: 21–60.

[15] Pfeiffer S, Williamson RF, Sealy JC, Smith DG, Snow MH. Stable dietary isotopes and mtDNA from Woodland period southern Ontario people: results from a tooth sampling protocol. J Archaeol Sci. 2014;42:334–345.

[16] Pfeiffer S, Sealey J, Williamson RF, Needs-Howarth S, Lesage L. Maize, fish, and deer: investigating dietary staples among ancestral Huron-Wendat villages, as documented from tooth samples. Amer Antiq. 2016; In press.

[17] Warrick GA. population history of the Huron-Petun, AD 500–1650. New York:Cambridge University Press; 2008.

[18] Dodd CF, Poulton DR, Lennox PA, Smith DG, Warrick GA. The Middle Ontario Iroquoian Stage In Ferris N., editor. The archaeology of Southern Ontario to AD. 1650;1990 pp. 321–59.

[19] MacDonald RI, Williamson RF. Sweat lodges and solidarity: The archaeology of the Hubbert site. Ont Archaeol. 2001; 71: 29–78.

[20] Williamson, RF, Steiss, DA. A history of Iroquoian burial practice. In Williamson, RF, Pfeiffer, S, editors Bones of the ancestors: the archaeology and osteobiography of the Moatfield Ossuary. Archaeological Survey of Canada Mercury Series Paper No. 163. Gatineau, Ontario:Canadian Museum of Civilization; 2003, pp. 89–132.

[21] Birch J. Coalescent communities: settlement aggregation and social integration in Iroquoian Ontario. Amer Antiq. 2012; 77: 646–670.

[22] Gramly RM. Richard Michael Deerskins and hunting territories: competition for a scarce resource of the Northeastern Woodlands. Amer Antiq. 1977; 42:601–605.

[23] Williamson RF. ‘Ontinontsiskiaj ondaon’ (The house of cut-off heads): The history and archaeology of northern Iroquoian trophy taking In Chacon RJ, Dye DH, editors. The taking and displaying of human body parts as trophies. New York:Springer Books; 2007 pp 190–221.

[24] Trigger BG. The children of Aataentsic: a history of the Huron people to 1660. McGill- Queen’s Montreal: University Press; 1976.

[25] Archaeological Services Inc. Report on the Stage 3–4 salvage excavation of the Alexandra site (AkGt-53) draft plan of subdivision SC-T20000001 (55T-00601) Geographic Township of Scarborough now in the City of Toronto, Ontario. Report on file, Toronto:Ontario Ministry of Culture; 2008

[26] Archaeological Services Inc. The archaeology of the Robb Site (AlGt-4): a report on the stage 4 mitigative excavation of the Angus Meadows Subdivision 19T-95030 (Revised) Part of Lot Concession 8 Town of Markham Regional Municipality of York, Ontario. Report on file, Toronto: The Ontario Ministry of Culture; 2010.

[27] Archaeological Services Inc. The stage 3–4 archaeological excavation of the Hope site (AlGv-199), draft plan of Subdivision 19T-02V07 and 19T-02V08, City of Vaughan, Regional Municipality of York, Ontario. Report on file, Toronto: The Ontario Ministry of Culture; 2011

[28] D. R. Poulton and Associates Inc. The 1992–1993 Stage 3–4 archaeological excavations of the Over site (AlGu-120), (W.P. 233-89-00), vol. 1. Report on file, Toronto:Ontario Ministry of Culture; 1996.

[29] Archaeological Services Inc. The Stage 4 Salvage excavation of the Baker site (AkGu-15) Lot 11 Concession 2 (WYS) Block 10 O.P.A. 400 Former Township of Vaughan, City of Vaughan, Regional Municipality of York, Ontario. Report on file, Toronto: The Ontario Ministry of Culture; 2006.

[30] Finlayson, WD. The 1975 and 1978 Rescue Excavations at the Draper Site: Introduction and Settlement Pattern, Archaeological Survey of Canada Mercury Series Paper No. 130, Ottawa: Canadian Museum of Civilization;1985.

[31] Finlayson, WD, Smith, DG, Spence, MW, Timmins, PA. The 1985 salvage excavations at the Keffer site: a license report. Report on file, Toronto: The Ontario Ministry of Culture; 1987.

[32] Ramsden C.N. The Kirche site. St John’s:Copetown Press; 1989.

[33] Archaeological Services Inc. The archaeology of the Mantle site (AlGt-334): report on the stage 3–4 mitigative excavation of part of Lot 22, Concession 9, Town of Whitchurch-Stouffville, Regional Municipality of York, Ontario. Report on file, Toronto: Ontario Ministry of Culture, Tourism and Sport; 2014.

[34] Ramsden, PG. Politics in a Huron village. In Keenlyside, DL, Pilon, J-L, editors, Painting the past with a broad brush: papers in honor of James Valliere Wright. Archaeological Survey of Canada, Mercury Series Paper No. 170. Gatineau: Canadian Museum of Civilization; 2009, pp. 299–318.

[35] Knight DA. Settlement patterns at the Ball site: A 17th century Huron village. Archaeol East N Am. 1987; 15:177–188.

[36] Ramsden PG. Saint Lawrence Iroquoians in the upper Trent River valley. Man in the Northeast. 1990; 39: 87–95.

[37] Warrick GA. European infectious disease and depopulation of the Wendat-Tionontate (Huron-Petun) World Archaeol. 2003; 35:258–275.

[38] Snow DR. Migration in prehistory: the northern Iroquoian case. Amer Antiq. 1995; 60:59–79.

[39] Voss B. The archaeology of ethnogenesis: race, sexuality, and identity in colonial San Francisco. Berkley: The University of California Press; 2008

[40] Voss B. What's new? Rethinking ethnogenesis in the archaeology of colonialism. Amer Antiq 2015; 80:655–670.

[41] Cippola C. Becoming Brothertown: Native American ethnogenesis and endurance in the modern world. Tucson: University of Arizona Press; 2013.

[42] Barth F. Introduction In Fredrik Barth F, editor. Ethnic groups and boundaries: the social organization of culture difference. London: George Allen & Unwin; 1969, pp. 9–38.

[43] Weber M. Economy and society: an outline of interpretive sociology. Berkley; The University of California Press; 1978.

[44] Blanton RE. Theories of ethnicity and the dynamics of ethnic change in multiethnic societies. Proc Nat Acad of Sci USA 2015; 112: 9176–9181. 25775584 10.1073/pnas.1421406112 PMC4522809

[45] Blanton RE, Fargher L. Collective action in the formation of pre-modern states. New York: Springer; 2008.

[46] Carballo DM, editor. Cooperation & collective action: archaeological perspectives. Boulder: University Press of Colorado; 2013.

[47] Wobst, HM. Stylistic behavior and information exchange. In Cleveland, CE, editor. For the director: research essays in honor of James B. Griffin. Anthropological Papers 61. Museum of Anthropology. Ann Arbor: University of Michigan;1977. pp. 317–342.

[48] Hill JB, Peeples MA, Huntley DL, Carmack HJ. Spatializing social network analysis in the late precontact US Southwest. Advan Archaeol Prac. 2015; 3:63–77.

[49] Clark, JJ, Huntley, DL. Hill, JB, Lyons PD. The Kayenta diaspora and Salado meta-identity in the late precontact US Southwest. In Card, JJ editor, The archaeology of hybrid material culture. Southern Illinois University Carbondale, Center for Archaeological Investigations, Occasional Paper No. 39. Carbondale: The Southern Illinois University Press; 2013. pp. 399–424.

[50] Hu D. Approaches to the Archaeology of Ethnogenesis: Past and Emergent Perspectives. J Archaeol Res. 2013; 21:371–402.

[51] Jones S. The archaeology of ethnicity: constructing identities in the past and present. London: Routledge; 1997.

[52] Insoll T, editor. The archaeology of identities: a reader. London, Routledge; 2006.

[53] Brumbach HJ. The history of the collared rim in the Finger Lakes, New York In Rieth CB, Hart JP, editors, Current research in New York archaeology: AD 700–1300. New York State Museum Record 3 Albany, New York State Education Department; 2011, pp 83–94.

[54] Taché K, Craig OE. Cooperative harvesting of aquatic resources and the beginning of pottery production in north-eastern North America. Antiquity. 2015;89(343):177–190.

[55] Tooker E. An ethnography of the Huron Indians, 1615–1649 Smithsonian Institution Bureau of American Ethnology Bulletin 190 Washington: U.S. Government Printing Office; 1964.

[56] Brown JK. Economic organization and the position of women among the Iroquois. Ethnohist 1970; 17:151–167.

[57] Lafitau JF. Moeurs des sauvages Ameriquains, comparees aux Moeurs des Premiers Temps. Four volumes. Paris: Saugrain l'aîné, 1724.

[58] Trigger BG. Iroquoian matriliny. Penn Archaeol. 1978; 48(1–2):55–65.

[59] Bowser BJ, Patton JQ. Domestic spaces as public places: an ethnoarchaeological case study of houses, gender, and politics in the Ecuadorian Amazon. J Archaeol Meth Theor. 2004 11:157–181.

[60] Bowser BJ. From pottery to politics: an ethnoarchaeological study of political factionalism, ethnicity, and domestic pottery style in the Ecuadorian Amazon. J Archaeol Meth Theor. 2000; 7:219–248.

[61] Chilton, E. A. Embodiments of choice: Native American ceramic diversity in the New England interior. Unpublished Ph.D. dissertation, Department of Anthropology. Amherst: University of Massachusetts; 1996.

[62] Carr C. A unified middle-range theory of artifact design In Carr C, Neitzel JE, editors, Style, society, and person: archaeological and ethnological perspectives. New York: Plenum Press; 1995, pp. 171–258.

[63] Steere BA. Swift Creek paddle designs as tattoos In Deter-Wolf A, Diaz-Granados C, editors. Drawing with great needles: ancient tattoo traditions of North America. Austin: The University of Texas Press; 2014, pp 73–94

[64] Engelbrecht, WE. A stylistic analysis of New York Iroquois pottery. Ph.D. dissertation, Department of Anthropology. Ann Arbor: University of Michigan; 1971.

[65] Engelbrecht WE. Appendix A: ceramic code and data In Sullivan L. E. (Ed.), Reanalyzing the Ripley site: earthworks and late prehistory on the Lake Erie plain. New York State Museum Bulletin 489 Albany: The University of the State of New York; 1996, pp. 129–153.

[66] Smith DG. Archaeological systematics and the analysis of Iroquoian ceramics: a case study from the Crawford Lake area, Ontario, Canada Bulletin 15 London, Ontario: London Museum of Archaeology;1997.

[67] Bursey JA. Prehistoric Huronia: Relative Chronology Through Ceramic Seriation. Ont Archaeol. 1993; 55:3–34.

[68] Kintigh, K. Tools for quantitative archaeology. Retrieved 26 May 2010 from http://tfqa.com/doc; 2010

[69] Smith B, Wilson JB. A consumer's guide to evenness indices. Oikos 1996; 76: 70–82.

[70] Brandes U, Wagner D. visone—analysis and visualization of social networks In Jünger M, Mutzel P editors, Graph drawing software. Springer-Verlag; 2004 pp 321–340.

[71] Nocaj A, Ortmann M, Brandes U. Untangling the hairballs of multi-centered, small-world online social media networks. J Graph Algorithms Appl 1984; 19(2): 595–618.

[72] Everett MG, Borgatti SP. Categorical attribute based centrality: E–I and G–F centrality. Soc Networks. 2012; 34: 562–569.

[73] Krackhardt D, Stern RN. Informal networks and organizational crises: an experimental simulation. Soc Psychol Quart 1988; 51:123–140.

[74] Borck L, Mills BJ, Peeples MA, Clark JJ. Are social networks survival networks? An example from the late pre-Hispanic US Southwest. J Archaeol Meth Theor. 2015 22: 33–57.

[75] Sokal R, Rohlf FJ. Biometry. New York:WH Freeman and Company;1995.

[76] Birch J, Wojtowicz RB, Pradzynski A, Pihl RH. Multi-scalar perspectives on Iroquioan ceramics: aggregation and integration in precontact Ontario In Jones EE, Creese JL, editors. Process and meaning in spatial archaeology: investigations into pre-Columbian Iroquoian space and place. Boulder: University Press of Colorado; 2016, In press.

[77] Brughmans T. Thinking through networks: a review of formal network methods in archaeology. J Archaeol Meth Theor. 2013; 20:623–662.

[78] Watts DJ., Strogatz SH. Collective dynamics of “small-world” networks. Nature, 1998; 393:440–442. 9623998 10.1038/30918

[79] Roscoe P. War, collective action, and the “evolution” of human polities In Carballo DM, editor, Cooperation and collective action: archaeological perspectives. Boulder:University of Colorado Press;2013, pp. 57–82

[80] Hegmon, M. Social dynamics of pottery style in the early Puebloan Southwest. Occasional Paper No. 5. Cortez, Colorado:Crow Canyon Archaeological Center; 1995.

