# Supplementary material for: Nation Building and Social Signaling in Southern Ontario: A.D. 1350–1650
Source: PLoS One. 2016 May 25;11(5):e0156178. doi: 10.1371/journal.pone.0156178 (PMC4880188; doi:10.1371/journal.pone.0156178)
Supplement: S1 Table — (PDF) [file pone.0156178.s002.pdf]

Table S1. Sources of data used in motif category coding for SNA.

Site	Number of Collars	Source of data for motif category coding
Alexandra	205	1
Alonzo	96	2
Ames	114	3
Antrex	746	4
Auger	67	3
Augoutenc	94	2
Aurora	120	5
Baker	134	6
Bark	103	7
Barker (Bain)	117	8
Barrie	270	9
Bathurst	108	10
Baumann	859	11
Benson	978	8
Bernault	37	3
Best	164	12
Bidmead	431	2
Black Creek	380	5
Boyle-Atkinson	123	13
Bradt	48	3
Campbell	174	14
Carson	285	2
Cedar Point	112	15
Charlebois	153	15
Chew	118	3
Christianson	388	16
Chypchar	146	14
Cooper	39	3
Copeland	1470	2
Coulter	382	17
Crawford Lake	118	14
Damiani	322	18
Deshambault	102	14
Draper	11359	19
Drumholm	52	20
Drury	27	3
Dunn 1	27	3
Dunsmore	173	21
Dykstra	57	22
Ellery	26	3
Emerson Springs	143	23
Farlain Lake	150	15

Finch	26	24
Forget	120	2
Fournier	914	2
Gibson	52	25
Glebe	104	26
Goodeve	75	2
Grandview	174	27
Gregor	105	28
Hamilton	197	29
Hanes	132	3
Haney-Cook	126	26
Hardrock	228	8
Heron	25	3
Hidden Spring	154	30
Hillier	121	31
Holly	559	32
Hood	72	33
Hope	174	34
Hubbert	93	35
Hunter's Oro 17	119	2
Ivan Elliot	124	3
Jackes	60	36
Jarrett-Lahmer	346	37
Joseph Picard	871	38
Keffer	5526	39
Kelly-Campbell	432	26
Kirche	892	8
Lalonde	125	2
Lawson	702	20
Le Caron	710	40
Lite	360	8
Logan	98	3
Lougheed	205	41
MacMurchy	227	26
Mantle	1575	42
McAllister	80	26
McGaw	43	43
McNair	506	44
Messenger	153	20
Milton	99	14
Molson	505	45
New	44	46
Nodwell	396	47
Nott	35	20
Orion Murphy-Goulding (O-M-G)	80	48, 49
Orr Lake	508	5

Parsons	169	50
Payne	245	51
Pengilly	55	3
Pipeline	386	14
Plater Martin	59	26
Pound	599	5
Pugh	204	11
Quackenbush	159	52
Raymond Reid	63	3
Rife	287	14
Risebrough	97	53,54
River	144	3
Robb	582	55
Robitaille	631	15
Rumney Bay	28	8
Second Lake	49	3
Seed-Barker	149	56
Serena	26	57
Sidey-Mackay	276	5
Snodden	120	58
Southwold	362	20
Spang	362	12
Starr	29	3
Train Farm	26	3
Trent-Foster	39	5
Unick	54	14
Van Eden	87	14
Vints	89	3
Walkington 2	100	59
Warminster	277	5
Waupoos	267	5
Webb	68	3
Wellington	135	60
White	104	26
Wiacek	152	2
Wilson	190	61
Woodbridge	288	5

SOURCES OF DATA FOR MOTIF CATEGORY CODING

1. Archaeological Services Inc. Report on the Stage 3-4 salvage excavation of the Alexandra site (AkGt-53) draft plan of subdivision SC-T20000001 (55T-00601) Geographic Township of Scarborough now in the City of Toronto, Ontario. Report on file, Toronto:Ontario Ministry of Culture, Tourism and Sport; 2008.
2. Bursey JA. Prehistoric Huronia: relative chronology through ceramic seriation. Ont Archaeol. 1993; 55:3–34.
3. Bursey, JA. Unpublished data used by permission.
4. Archaeological Services Inc. Report on salvage excavations of the Antrex site (AjGv-38), City of Mississuagua, Regional Municipality of Peel, Ontario. Report on file, Toronto:Ontario Ministry of Culture; 2010.
5. Canadian Museum of History, Ottawa, Ontario. Richard S. MacNeish fonds, folder: “Ontario pottery types research notes”[195-]). Boxes 25 and 26, MacNeish.
6. Archaeological Services Inc. The Stage 4 Salvage excavation of the Baker site (AkGu-15) Lot 11 Concession 2 (WYS) Block 10 O.P.A. 400 Former Township of Vaughan, City of Vaughan, Regional Municipality of York, Ontario. Report on file, Toronto: The Ontario Ministry of Culture, Tourism and Sport; 2006.
7. Sutton, RE. Middle and Late Iroquoian occupations of the middle Trent valley. Masters thesis, Department of Anthropology. Hamilton, Ontario: McMaster University; 1989.
8. Ramsden, PJ. Unpublished coding forms, used by permission.
9. Sutton, RE. The Barrie site: a pioneering Iroquoian village located in Simcoe County, Ontario. Ont Archaeol. 1999; 67:40–85.
10. Archaeological Services Inc. Stage 4 archaeological salvage excavation and monitoring of Site BaGv-75, Bathurst Street Reconstruction, Lot 10, Concession 2, Township of King and Lots 103-104, Concession 1, Town of East Gwillimbury Regional Municipality of York. Report on file, Toronto:Ontario Ministry of Culture; 2016
11. Coded from collections at the Huronia Museum 549 Little Lake Park Rd. Midland Ontario.L4R 4P4 . 705 526-2844
12. Birch, J, Wojtowicz, RB, Pradzynski, A, Pihl, RH. Multi-scalar perspectives on Iroquioan ceramics: aggregation and integration in precontact Ontario. In Jones, EE, Creese, JL, editors. Process and meaning in spatial archaeology: investigations into pre-Columbian Iroquoian space and place. Boulder: University Press of Colorado; 2016, In press.
13. BAIF Associates, Inc. The 1984 salvage excavations at the Boyle-Atkinson site (AlGu-1), Town of Richmond Hill, Ontario. Report on file, Toronto: The Ontario Ministry of Culture, Tourism and Sport; 1984.
14. Smith, DG. Archaeological systematics and the analysis of Iroquoian ceramics: a case study from the Crawford Lake area, Ontario, Canada. Bulletin 15. London, Ontario: London Museum of Archaeology;1997
15. Latta, MA. The Iroquoian cultures of Huronia: a study of acculturation through archaeology. Unpublished Ph.D. dissertation, Department of Anthropology, University of Toronto; 1976.
16. Fitzgerald, RW. Lest the beaver run loose: The early 17th century Christianson site and trends in historic Neutral archaeology. Masters thesis, Department of Anthropology, Hamilton, Ontario: McMaster University; 1981.
17. Damkjar, E. Unpublished coding forms used by permission.

18. Archaeological Services Inc. Report on the stage 3–4 mitigative excavation of the Damiani site (AlGv-231). City of Vaughan, Regional Municipality of York, Ontario. Report on file, Toronto: Ontario Ministry of Culture, Tourism and Sport; 2012.
19. Phil, RH. Final report on the Draper rim collection: 1975/1978 excavated samples. Unpublished report available at Archaeological Services, Inc, Toronto; 1984.
20. Smith, DG. An analytical approach to the seriation of Iroquoian pottery. Unpublished M.A. thesis, Department of Anthropology, Montreal: McGill University; 1980
21. Robertson, DA, Williamson, RF. The archaeology of the Dunsmore site: 15th-century community transformations in southern Ontario. Canadian J Archaeol. 2003; 27: 1–61.
22. Archaeological Services Inc. The archaeology of the Dykstra site (BbGw-5), a report on stage 4 excavations at the Holly secondary planning area (43T-92026), part of the northwest half of Lot 2, Concession 12, City of Barrie, Simcoe County, Ontario. Report on file, Toronto: The Ontario Ministry of Culture, Tourism and Sport; 2006.
23. Hawkins, AL. Report on the 2004 investigations at the Emerson Springs Village (AkGx-5), Town of Halton Hills, Ontario Under License P081-002 and P081-004. Report on file, Toronto: The Ontario Ministry of Culture, Tourism and Sport; 2004.
24. Pihl, RH, Thomas, SC. The Finch site: a late Iroquoian special purpose site on West Catfish Creek. Ont Archaeol, 1987; 63:37–84.
25. Archaeological Services, Inc. Stage 3 Archaeological Assessment of the Gibson Site (BcGo-14), Gibson Subdivision, Part of Lot 18, Concession 10, Township of Smith-Ennismore-Lakefield, County of Peterborough, Ontario. Report on file, Toronto: The Ontario Ministry of Culture, Tourism and Sport; 2008.
26. Coded from collections in the Charles Garrad Collection, Archaeological Services Inc., 528 Bathurst Street, Toronto, On. M5S 2P9.
27. Williamson RF, Thomas SC. The archaeology of the Grandview site: a fifteenth-century Iroquoian community on the north shore of Lake Ontario. Arch Notes 2003; 8(5): 5–48.
28. Archaeological Resource Associates. Stage 4 archaeological assessment Gregor site (BbGw-16) north part of Lot 1, Concession 11 City of Barrie, County of Simcoe former Township of Innisfil 43T-92010. Report on file, Toronto: The Ontario Ministry of Culture, Tourism and Sport; 2003.
29. Lennox, PA. The Hamilton site: a late historic Neutral town. Masters thesis, Department of Anthropology, Hamilton, Ontario: McMaster University; 1977.
30. Archaeological Services Inc. The archaeology of the Hidden Spring site (AlGu-368): stage 4 salvage excavation of the Hidden Spring Site, Oxford West Subdivision Development, part of Lots 13-16 and 37-40, Registered Plan 1931, Town of Richmond Hill, Regional Municipality of York, Ontario. Report on file, Toronto: The Ontario Ministry of Culture, Tourism and Sport; 2010.
31. Coded from collections at the Canadian Museum of History, 100 Rue Laurier, Gatineau, QC K1A 0M8(819) 776-7000
32. Archaeological Services Inc. The archaeology of the Holly site (BcGw-58), stage 4 salvage excavation of the Holly site, Dykstra Subdivision, Holly Secondary Planning Area (43T-92026), part of Northeast Half of Lot 2, Concession 12, City of Barrie, Simncoe County, Ontario. Report on file, Toronto: The Ontario Ministry of Culture, Tourism and Sport; 2009.
33. Fitzgerald, WR. The Hood site: longhouse burials in an historic Neutral village. Ont Archaeol. 1979; 32: 43–60.

34. Archaeological Services Inc. The stage 3-4 archaeological excavation of the Hope site (AlGv-199), draft plan of Subdivision 19T-02V07 and 19T-02V08, City of Vaughan, Regional Municipality of York, Ontario. Report on file, Toronto: The Ontario Ministry of Culture, Tourism and Sport; 2011
35. MacDonald, RI, Williamson, RF. Sweat lodges and solidarity: the archaeology of the Hubbert site. Ont Archaeol. 2001; 71: 29–78.
36. Noble, WC. The Jackes (Eglinton) site: another facet of southern Huron development in the Toronto region. Ont Archaeol. 1974; 22:3–31.
37. Archaeological Services Inc. The 1999 stage 2-4 assessment of part of the Jarrett-Lahmer site (AlGv-18), Lot 17, Concession 5 (WYS), former Township of Vaughan, City of Vaughan, Regional Municipality of York, Ontario. Report on file, Toronto: The Ontario Ministry of Culture, Tourism and Sport; 2005.
38. Archaeological Services Inc. The archaeology of the Picard site (AlGs-376): a report on the stage 3&4 mitigative excavation of the Joseph Picard site (AlGs-376) Highway 407 East, Lot 32, Concession VI, Whitby Township, Former Ontario County Regional Municipality of Durham, Ontario. Report on file, Toronto: The Ontario Ministry of Culture, Tourism and Sport; 2016.
39. Coded from collections at the Museum of Ontario Archaeology, 1600 Attawandaron Rd. London, Ontario. N6G 3M6
40. Cameron, KL. Pots and people: an examination of the relationships between production, function, and distribution at the Le Caron site, Simcoe County, Ontario. Ph.D. dissertation, Department of Anthropology, Durham, Ontario: Trent University; 2011.
41. Finlayson, WD. Stage 3 & 4 archaeological assessment of he Lougheed site (BbGw-13), north ½ of Lot 1, Concession 11, City of Barrie, County of Simcoe, former Township if Innisfil. Report on file, Toronto: Ontario Ministry of Culture, Tourism and Sport; 2003.
42. Archaeological Services Inc. The archaeology of the Mantle site (AlGt-334): report on the stage 3-4 mitigative excavation of part of Lot 22, Concession 9, Town of Whitchurch-Stouffville, Regional Municipality of York, Ontario. Report on file, Toronto: Ontario Ministry of Culture, Tourism and Sport; 2014.
43. Archaeological Services Inc. McGaw site (AlGu-88) archaeological interpretive program results of the 2003 field season. Report on file, Toronto: Ontario Ministry of Culture; 2003
44. Archaeological Services Inc. The Archaeology of the McNair Site (AlGu-8), A Report on the Stage 3-4 Mitigative Excavation of the McNair Site (AlGu-8), Block 12, OPA 400, Draft Plan of Subdivision 19T-89124 (Major Bob Farms Inc.) And Draft Plan of Subdivision 19T-99V-08 (Andridge Homes Limited Lands) Part of Lots 24 and 25, Concession 2in the City of Vaughan Regional Municipality of York, Ontario. Report on file, Toronto: The Ontario Ministry of Culture, Tourism and Sport; 2012.
45. Lennox PA. The Molson site: an early seventeenth century, First Nations settlement, Simcoe County, Ontario. Research Bulletin 18. London, Ontario: London Museum of Archaeology; 2000.
46. Archaeological Services Inc. Stage 4 archaeological excavation of the New site (AlGt-36), Ibrans Box Grove Property, draft plan of Subdivision 19TM-04001, Town of Markham, Regional Municipality of York, Ontario. Report on file, Toronto: Ontario Ministry of Culture, Tourism and Sport; 2010.
47. Wright JV. The Nodwell site. Mercury Series No. 22. Ottawa: National Museums of Canada; 1974.

48. Archaeological Services Inc. The stage 4 salvage excavation of the Orion site (AlGu-45) Lot 56, Concession 1 W.Y.S, Town of Richmond Hill, Regional Municipality of York, Ontario. Report on file, Toronto: The Ontario Ministry of Culture, Tourism and Sport; 2008.
49. Archaeological Services Inc. Final report on the archaeological salvage excavation of the Murphy-Goulding site (AlGu-3), Town of Richmond Hill, Regional Municipality of York. Report on file, Toronto: Ontario Ministry of Culture, Tourism and Sport; 1998.
50. Williamson, RF, Powis, TG. Parsons site ceramic vessels. Ont Archaeol. 1998; 65/66:53–71.
51. Emerson JN. Understanding Iroquois pottery in Ontario: a rethinking. Mississauga: Ontario Archaeological Society; 1968.
52. Coded from collections at the Anthropology Department, Trent University, Peterborough, Ontario.
53. Coded from collections at the Department of Anthropology, University of Toronto, St. George Campus. 19 Russel St. Toronto M5S 2S2
54. Coded from collections at New World Archaeology, Royal Ontario Museum, 100 Queens Park, Toronto, ON M5S 2C6.
55. Archaeological Services Inc. The archaeology of the Robb Site (AlGt-4): a report on the stage 4 mitigative excavation of the Angus Meadows Subdivision 19T-95030 (Revised) Part of Lot Concession 8 Town of Markham Regional Municipality of York, Ontario. Report on file, Toronto: The Ontario Ministry of Culture, Tourism and Sport; 2010.
56. Burgar, RWC. The Seed-Barker project: 1988. Report submitted to the Minister of Culture and Communications in fulfillment of licensing requirement for license no. 88-30. (1989)
57. Archaeological Services Inc. Report on stage 4 excavation of the Serena site (AhGx-274), Allison Estates, Subdivision (25T-91014), City of Hamilton, Regional Municipality of Hamilton, Ontario. Report on file, Toronto: The Ontario Ministry of Culture, Tourism and Sport; 2004.
58. Coded from collections at York North Archaeological Services 1264 Bathurst St, Peterborough, ON K9H 6X8 (705) 742-7301.
59. Archaeological Services Inc. The archaeology of the Walkington 2 site (AlGu-341), draft plan of Subdivision 19T-95066 (revised), part of Lots 16 and 17, Con. 2, City of Vaughan, R.M. of York, Ontario. Report on file, Toronto: The Ontario Ministry of Culture, Tourism and Sport; 2011.
60. Archaeological Services Inc. The archaeology of the Wellington site (BcGw-55), a report on the stage 4 salvage excavations of the Wellington site, Holly Secondary Planning Area (43T-92023), part of the east half of Lot 3, Concession 12, City of Barrie, Simcoe County, Ontario. Report on file, Toronto: The Ontario Ministry of Culture, Tourism and Sport; 2005.
61. Sutton, RE. Hidden amidst the hills: Middle and Late Iroquoian occupations in the middle Trent Valley. Dundas, Ontario: Copetown Press;1990.
